# Species composition, diversity, and distribution of the genus *Ulva* along the coast of Jeju Island, Korea based on molecular phylogenetic analysis

**DOI:** 10.1371/journal.pone.0219958

**Published:** 2019-07-23

**Authors:** Ji Hyoun Kang, Ji Eun Jang, Jae Hwan Kim, Seo Yeon Byeon, Sangil Kim, Sun Kyeong Choi, Yun Hee Kang, Sang Rul Park, Hyuk Je Lee

**Affiliations:** 1 Korean Entomological Institute, College of Life Sciences and Biotechnology, Korea University, Seoul, Korea; 2 Molecular Ecology and Evolution Laboratory, Department of Biological Science, Sangji University, Wonju, Korea; 3 Gencube, Gimpo, Korea; 4 Ocean Climate and Ecological Research Division, National Institute of Fisheries Science, Busan, Korea; 5 Estuarine and Coastal Ecology Laboratory, Department of Marine Life Sciences, Jeju National University, Jeju, Korea; Macquarie University, AUSTRALIA

## Abstract

Species diversity in the genus *Ulva* remains understudied worldwide. Using molecular analyses we investigated the species composition, diversity, distribution, and relative frequencies of the genus *Ulva* along the entire coast of Jeju Island, off the southern tip of Korea. Species identification was performed for 215 samples collected from 23 sites, based on comprehensive phylogenetic and model-based species delimitation analyses using the sequences of two molecular markers, chloroplast elongation factor Tu (*tuf*A) and nuclear rDNA internal transcribed spacer (ITS). We identified 193 specimens as nine *Ulva* species, 14 specimens as *Blidingia* spp., and eight samples undetermined, based on the combined analysis of *tuf*A and ITS phylogenies. Two model-based approaches generally supported nine groups of *Ulva* species. Previously documented species complex, such as *U*. *ohnoi−U*. *spinulosa* and *U*. *procera−U*. *linza* showed discordant relationships between the two phylogenies. The occurrence of *U*. *torta* on Jeju Island was first observed, despite its existence on the mainland previously reported. *Ulva australis* [16 of 23 sites; 34.4% (relative frequency)], *U*. *ohnoi* (16; 21.9%), and *U*. *procera* (11; 14%) were found to be the predominant species. Our study highlights that molecular analysis is critical for species delimitation in the genus *Ulva* and provides fundamental information for an understanding of green-tide assemblages on the “biological hotspot” coastal ecosystem, Jeju Island in Korea. This study will also help to monitor and manage local green tides at the areas that are currently encountering rapid climate changes.

## Introduction

The marine green macroalgal genus *Ulva*, especially notorious for green tide formation, is comprised of approximately 100 species, of which 18 species have so far been recorded from Korea [1–4]. Although species of *Ulva* have a simple multicellular thallus structure, they show a wide range of complex shapes due to phenotypic plasticity and differences in morphogenesis [5, 6]. Therefore, morphology based species identification in the genus *Ulva* often involves taxonomic errors due to intraspecific variation [4–7]. In Korea, previous taxonomic classifications for *Ulva* have mostly consisted of morphological investigations, therefore the reported number of species might be over- or under-estimated [2, 3, 8, 9]. Relatively little attention has been paid to molecular-based ecological surveys for species identification of the genus *Ulva* from this country [but see 4, 10]. There is a need to accurately identify native and non-native species of *Ulva* because some have been implicated in causing green tides [10]. A thorough documentation of the biodiversity of the Ulvalean native flora using molecular tools is essential to understanding and managing algal growth as well as marine ecology in Korea.

**Fig 1:**
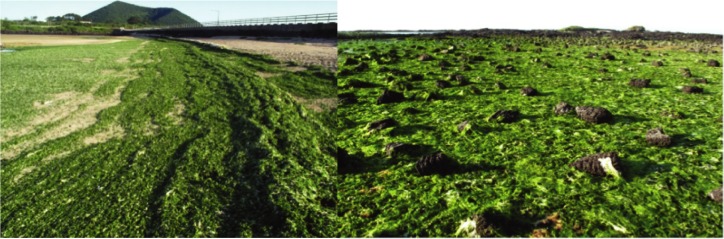
Green algal *Ulva* mats on the shoreline of the Hado Beach (site 13B) on Jeju Island in Korea. Photos were taken on July 2013.

In recent years, green algal massive growth forming “green mats” have been frequently detected all year round along the shorelines on Jeju Island, off the southernmost region of Korea (Fig 1) [11]. Such events have been reported from the southern coast of the mainland [12, 13]. These phenomena have been considered as one of the largest problems in maintaining “healthy” coastal ecosystems in Jeju Island [10], being well known as a biological hotspot [14] and also as a popular holiday destination. Nevertheless, what species are major constituents for such “green carpets”, and the species diversity and distribution of the genus *Ulva* at an entire-island scale remain largely unknown.

In the present study, we aim to determine the species composition and diversity, geographic distribution, and relative frequencies of *Ulva* species along the entire coast of Jeju Island, a pristine habitat and hotspot for biodiversity. To this end, we conducted three different phylogenetic analyses [neighbor-joining (NJ), maximum-likelihood (ML) and Bayesian inference (BI)] on a total of 215 samples collected from 23 sites using two sets of molecular markers, including nuclear (nu) DNA, internal transcribed spacer 1 (ITS1), 5.8S ribosomal RNA, ITS2 region (ITS1-5.8S-ITS2; ITS), and chloroplast (cp) DNA encoded elongation factor Tu (*tuf*A). Additionally, two model-based species delimitation approaches, such as Automatic Barcode Gap Discovery (ABGD) [15] and Generalized Mixed Yule Coalescent (GMYC) [16] were conducted to validate the results of our phylogenetic analyses. One of the goals of this study is to verify and discuss the accuracy and reliability of these two markers for the species delimitation of *Ulva*. The results of our study will provide fundamental information on the green-tide forming *Ulva* species and lay the groundwork for future research on managing and mitigating local green tides in the “biological hotspot” coastal ecosystem, Jeju Island, in Korea.

## Materials and methods

### Study area and sample collection

**Fig 2:**
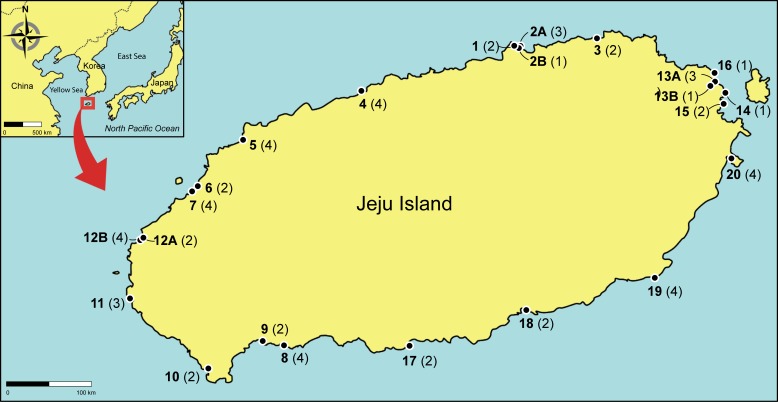
Map of 23 collection sites along the coast of Jeju Island in Korea. The collection site numbers are given in bold. Those numbers cross-reference to Table 1 and S1 Table. Names of these sampling sites are given in S1 Table. The number of species found in each site, based on our molecular analyses is shown within parentheses.

No specific permission to collect samples was required at the study sites, and the field research did not involve endangered or protected species. This study was conducted along the coastline of Jeju Island, which is located approximately 150–200 km off the southern coast of the Korean Peninsula (Fig 2). Jeju Island is known as one of the fastest warming regions worldwide; the sea surface temperature around this region has risen by 1.6°C–2.1°C over the last 100 years [17]. This island has been suggested as an ecosystem supporting high biodiversity, particularly for marine benthic species [14]. The tidal regime is semi-diurnal with a maximum tidal range of about 2.7 m during spring tides (Tide Tables for the Coast of Korea, National Oceanographic Research Institute of Korea).

We collected 215 specimens, morphologically identified as *Ulva* species, from 23 sites along the entire coast of Jeju Island in April 2015 (Table 1; Fig 2; S1 Table). All samples were collected at 2–3 meter intervals of each other within sites, and these sampling distances were maintained across sites. More than 2 individuals per morphotypes were collected from each of the 23 sites by considering different species based on morphology. However, we could not perform detailed morphological assessments of the collected specimens, partly due to the fact that they were not always in intact shape. Detailed information for collection sites and number of species are shown in S1 Table.

**Table 1:** Species composition, diversity and relative frequency of the genus *Ulva* and *Blidingia* spp. along the coast of Jeju Island in Korea, based on our molecular phylogenetic analyses.

Species	Nr. of specimens (%)	Nr. of site	Sampling sites
***U*. *australis* (= *U*.*pertusa*)**^**a**^	74 (34.4)	16	1,2a,3,4,5,6,7,8,10,12a,12b,13a,15,16,19,20
***U*. *compressa***	4 (1.9)	2	6,15
***U*. *californica***	13 (6.0)	6	1,4,9,13A, 15,20,
***U*. *flexuosa***	8 (3.7)	5	2a, 11, 12B, 19, 20
***U*. *laetevirens* (*= U*.*rigida*)**^**a**^	4 (1.9)	2	11,19
***U*. *ohnoi* (*U*. *spinulosa*)**^**b**^	47 (21.9)	16	1,2a,2b,5,7,8,9,10,12a,12b,13b,14,17,18,19,20
***U*. *procera* (*U*. *linza*)**^**b**^	30 (14.0)	11	2a,3,4,5,6,7,8,11,13a,17,20
***U*. *torta***	4 (1.9)	2	12b,8
***U*. *arasakii***	9 (4.2)	3	4,5,7
***Blidingia* spp.**	14 (6.5)	7	4,5,7,9,11,13a,16
**Discordant species**	8 (3.7)	6	2a,3,4,7,8, 15
**Total**	215	23	

The analyses were performed for the sampled 215 specimens from 23 sites using two molecular markers (chloroplast DNA *tuf*A and nuclear DNA ITS). Discordant species (e.g., unidentified) were defined when two gene phylogenies suggested different species.

^a^Synonyms

^b^Species complexes

### Genomic DNA extraction, PCR, and sequencing

Collected specimens were washed with freshwater, patted dry, and raked to remove epiphytes if present. Leaf tissue was dried at 60°C for 24 h and pulverized using TissueLyserII (QIAGEN, USA). Powdered tissue samples were transferred to a 1.5-ml microcentrifuge tube with silica gel and stored at −20°C until genetic analysis. Genomic DNA was extracted using a DNeasy Plant Mini Kit (QIAGEN), according to the manufacturer’s protocol. Genomic DNA was quantified using Qubit 2.0 Fluorometer (Invitrogen, USA).

Polymerase chain reaction (PCR) was performed to amplify cpDNA *tuf*A (813 bp) and nuDNA ITS regions (732 bp) in a reaction volume of 20 μl. The primer TufGF4: 5'-GGN GCN GCN CAA ATG GAY GG-3' and tufAR: 5'-CCT TCN CGA ATM GCR AAW CGC-3' were used for the *tuf*A [18] and the primer 18S150F: 5'-TCT TTG AAA CCG TAT CGT GA-3' and ENT26SA: 5'-GCT TAT TGA TAT GCT TAA GTT CAG CGG GT-3' were used for the ITS [19]. PCR conditions were as follows: an initial denaturation at 94°C for 1 min; followed by 35 cycles of 30 s at 94°C, 30 s at 50°C–54°C, and 1–2 min at 72°C; and a final extension step of 72°C for 10 min. The PCR products were visualized on 1% agarose gels, and PCR products were purified and then sequenced in both directions by Macrogen Inc. Sequencing (Korea) using an ABI PRISM 3130xl genetic analyzer (Applied Biosystems, USA). All obtained sequences were deposited in GenBank nucleotide sequence database under accession Nos. (*tuf*A: MK992043-MK992249, ITS: MN069870-MN070070).

### Phylogenetic analyses for species identification

The capacity of species identification by phylogenetic analysis depends on the accuracy of reference sequences. Sequences designation for species in GenBank database could be inaccurate due to taxonomical errors resulting from high levels of morphological variation aroused by phenotype plasticity, which is quite common in the genus *Ulva* [5, 6]. Thus, we set several criteria to select the reference sequences for *Ulva* species to avoid false positive species identification and exclude ambiguous sequences from the GenBank database. To choose reference sequences for our phylogenetic analyses, we retrieved 437 *tuf*A gene sequences of *Ulva* species available from the database and reconstructed a NJ tree along with three sequences of *Blidingia* spp. (order Ulvales, family Kornmanniaceae) as an outgroup using MEGA7.0.14 [20] under the complete deletion option with 1000 bootstrap replicates. A total of 44 sequences representing 19 *Ulva* species were finally chosen as reference sequences by considering the selection criteria that one or two sequences were selected if conspecific sequences form a strong monophyly, and sequences were discarded if they show ambiguous phylogenetic positions, such as clustering with distantly related species.

A total of 262 sequences containing 215 sequences from the study specimens with 47 reference sequences (44 for 19 *Ulva* species, plus 3 for *Blidingia* spp.) were used to reconstruct the phylogenetic tree of the *tuf*A gene (S1 Table). All sequences were aligned using ClustalW multiple sequences alignment package [21], which is implemented in BioEdit 7.1.9 [22]. We conducted a NJ analysis with 1000 bootstrap replicates in MEGA. ML analysis was conducted under the GTR+G model with 1000 bootstrap replicates using RAxML Web-Servers [23] and BI, implemented in MrBayes 3.2 [24], was performed with HKY+G+I chosen as the best-fit substitution model by AICc with jModeltest 2.1.7 [25]. The MCMC run was set for 10,000,000 generations with four chains, and the first 25% of the samples were discarded as the burn-in.

For the ITS region, a total of 243 sequences containing 210 sequences from the sampled specimens (after excluding five specimens with a poor quality of sequences) with 33 reference sequences (30 for 27 *Ulva* species, plus three for *Blidingia* spp.) retrieved from the GenBank database were used for phylogenetic analyses (S1 Table). Sequence alignments and phylogenetic analyses based on NJ, ML and BI methods were performed as for *tuf*A phylogeny. Ambiguously aligned regions for the ITS, due to high levels of nucleotide diversity, were excluded for the phylogenetic analyses. The HKY+G substitution model was chosen for the 732 bp of the ITS region.

Species were first determined based on phylogeny of each gene separately, when the study specimens formed a monophyly (Table 1; S1 Table). For each gene, we chose a “species” delimitated by a majority of inferences, in cases of discordance found among three phylogenetic inferences (NJ, ML, and BI). We defined species as “discordant species” or “unidentified” when two gene phylogenies suggested different species. When more than one species in the reference sequences showed monophyletic relationships with the study specimens, we chose those as “species”, which had the lowest sequence divergence (K2P) with the study specimens.

### Model-based species delimitation analyses

**Fig 3:**
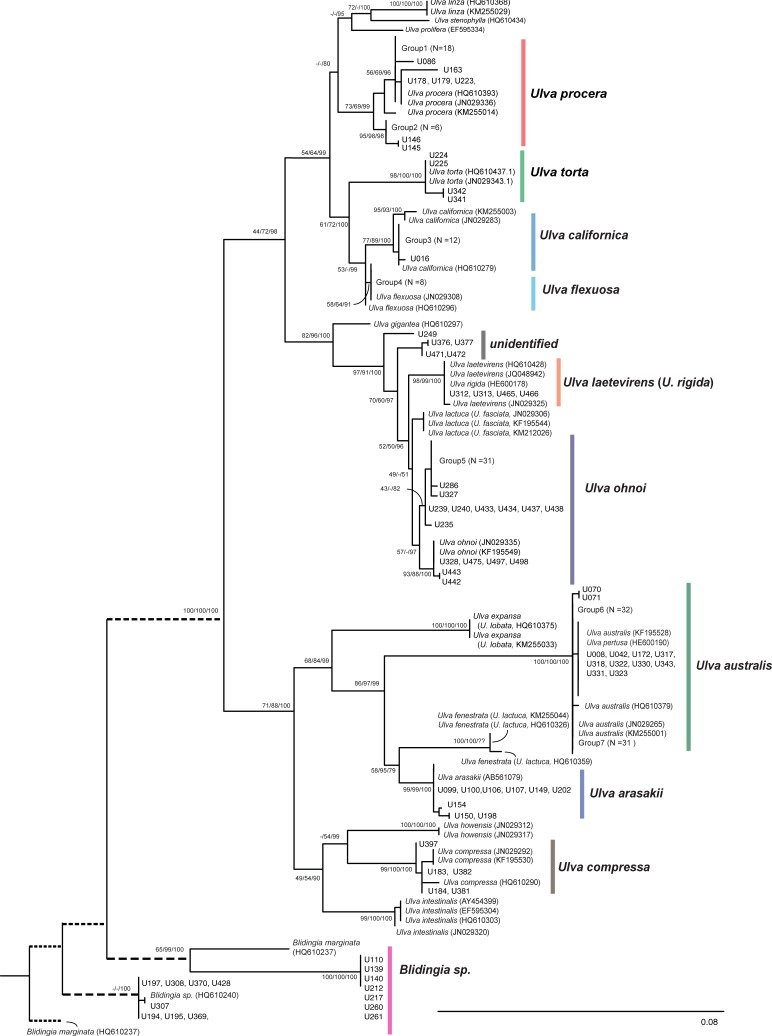
Maximum likelihood (ML) phylogram based on 262 *tuf*A sequences of *Ulva* and *Blidingia* species. Numbers indicate bootstrap values for maximum likelihood and neighbor-joining, and Bayesian posterior probabilities, respectively. The truncated branches are displayed with dashed lines. Sample IDs (e.g., U001) for each specimen collected in this study (cross-reference to S1 Table) are shown and GenBank accession numbers are included for reference sequences. A large number of specimens found within a particular clade are marked as “Group” with the total number of specimens [e.g., Group6 (N = 32)]. Species names of *U*. *lactuca*, *U*. *expansa*, and *U*. *fenestrata* are updated based on recently revised taxonomic status using genetic analysis of the holotype specimens [30]. Originally attributed names from GenBank are shown in parenthesis.

**Fig 4:**
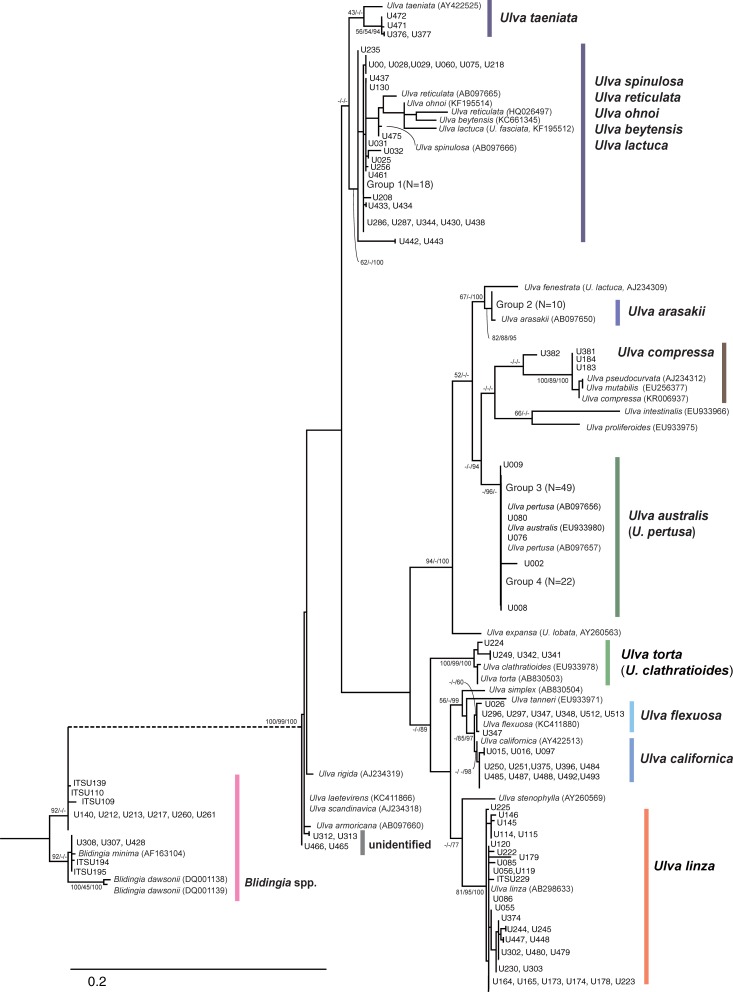
Maximum likelihood (ML) phylogram based on 243 ITS sequences of *Ulva* and *Blidingia* species. Numbers indicate bootstrap values for maximum likelihood and neighbor-joining, and Bayesian posterior probabilities, respectively. The truncated branches are displayed with dashed lines. Sample IDs (e.g., U001) for each specimen collected in this study (cross-reference to S1 Table) are shown and GenBank accession numbers are included for reference sequences. A large number of specimens found within a clade are marked as “Group” with the total number of specimens. Species names of *U*. *lactuca*, *U*. *expansa*, and *U*. *fenestrata* are updated based on recently revised taxonomic status using genetic analysis of the holotype specimens [30]. Originally attributed names from GenBank are shown in parenthesis.

We performed two different algorithmic species delimitation methods, ABGD and GMYC, to validate the results of the phylogeny-based species identification (Table 2; Figs 3 and 4). The ABGD analyses were run on the web-interface (https://wwwabi.snv.jussieu.fr/public/abgd/abgdweb.html) with default settings for both markers with an exception of the Kimura K80 distance model and three relative gap widths (X) (X = 1.0, 1.5, 2.0) applied. Values for the prior intraspecific diversity (*P*) was set from 0.001 to 0.1 (S2 Table).

**Table 2:** The number of groups (clusters) for each species obtained from ABGD (A) and GMYC (B) analyses inferred from chloroplast (*tuf*A) and nuclear (ITS) markers. X: relative gap width, *P*: prior intraspecific divergence.

Species identified by phylogenetic analyses (ML, BI, ML)	(A) ABGD				(B) GMYC	
	*tuf*A	*tuf*A	ITS	ITS	*tuf*A	ITS
	X = 1.0	X = 1.5	X = 1.0	X = 1.5		
	*P* = 0.0017	*P* = 0.0077	*P* = 0.0077	*P* = 0.0129		
*U*. *australis* (= *U*.*pertusa*)^a^	1	1	1	1	1	1
*U*. *arasakii*	1	1	1	1	1	
*U*. *compressa*	1	1	1	1	1	
*U*. *californica*	2	1	1	1	1	1
*U*. *flexuosa*	1				1	
*U*. *torta*	2		1	1	1	
*U*. *procera* (*U*. *linza*)^b^	5	1	1	1	1	
*U*. *laetevirens* (*= U*.*rigida*)^a^	1	1	1	1	1	1
*U*. *ohnoi* (*U*. *spinulosa*)^b^	2		1	1	1	
*Blidingia* spp.	4	4	3	3	not included	not included
Non sampled species groups (sequences from GenBank database only)	11	7	11	11	4	-
Total number of groups (clusters)	31 groups	17 groups	22 groups	22 groups	13 clusters	3 clusters
**Total number of groups (clusters) of *Ulva* specimens analyzed in this study**	**16 groups**	**6 groups**	**8 groups**	**8 groups**	**9** **clusters**	**3** **clusters**

Prior to model-based GMYC analyses, sequences were collapsed into unique haplotypes using DnaSP v5 [26]. For the GMYC analyses, ultrametric trees for each *tuf*A and ITS were generated using Bayesian inference implemented in BEAST v.2.5.2 [27]. Branch lengths were estimated assuming strict clock and Yule models as a tree prior. Markov Chains Monte Carlo (MCMC) was run for five million generations, sampling 1000 trees. Runs were inspected for convergence using Tracer v. 1.7 [28] and trees were summarized with a 25% burn-in. GMYC analyses were run with a single threshold using SPLITS package in R [29].

## Results

### Sequence analyses

A total of 262 *tuf*A (including 47 reference sequences) and 243 ITS sequences (33 reference sequences) were analyzed for species delimitation of 215 specimens collected across the Jeju Island coast (Table 1; Figs 3 and 4; S1 Table). The *tuf*A sequence alignment had a length of 813 bp with 285 variable nucleotide sites, of which 261 were parsimony-informative sites and 24 were singleton sites. The ITS region alignment included 732 bp with 261 variable sites, of which 202 were parsimony-informative sites and 59 were singleton sites. Estimates of the average sequence divergence (K2P) were 8.8% (±0.7%) for *tuf*A and 6.4% (±0.8%) for ITS. We also estimated pairwise distance (K2P) among reference sequences retrieved from the GenBank database for *Ulva* species. Overall mean distance among *Ulva* species for *tuf*A (44 sequences) and ITS (30 sequences) were 5.9% (±0.6%) and 8.7% (±0.9%), respectively. When we found a very low level of sequence divergence or no genetic variation between species, we considered them as the same species (i.e., synonym) in cases of both genes.

### Species diversity, composition, and relative frequencies based on phylogenetic analyses

In a total of 215 specimens, we identified 193 specimens as nine *Ulva* species, 14 specimens as *Blidingia* spp., and eight samples undetermined (e.g., discordance between two phylogenies), based on the comprehensive phylogenetic analyses using *tuf*A and ITS (Table 1; Figs 3 and 4). In the *tuf*A gene tree, nine clades were clustered with reference sequences of *U*. *procera*, *U*. *torta*, *U*. *californica*, *U*. *flexuosa*, *U*. *laetevirens*, *U*. *ohnoi*, *U*. *australis*, *U*. *arasakii*, and *U*. *compressa*. Estimates of average intraspecific divergence for the nine *Ulva* species ranged from 0.03% [*U*. *flexuosa* (N = 10)] to 0.54% [*U*. *procera* (N = 34)]. The highest level of interspecific divergence (11%) was found between *U*. *australis* and *U*. *torta*, whereas the lowest interspecific divergence was found between *U*. *laetevirens* and *U*. *rigida* (0.0%), *U*. *australis* and *U*. *pertusa* (0.1%), and *U*. *fasciata* (*U*. *lactuca* [30]) and *U*. *ohnoi* (0.7%).

ITS phylogeny revealed the same number of nine *Ulva* species, but species composition was slightly different from *tuf*A. Specimens clearly identified as *U*. *ohnoi* (N = 47) from the *tuf*A phylogeny formed monophyletic groups with six different species: *U*. *ohnoi*, *U*. *reticulata*, *U*. *beytensis*, *U*. *fasciata* (*U*. *lactuca* [30]), *U*. *spinulosa*, and *U*. *taeniata* in ITS phylogeny (Fig 4). Although internal relationships among these six species were not clearly resolved in all three phylogenetic analyses, the lowest divergence (0%) of specimens in the clade was found with *U*. *spinulosa* (Fig 4). Specimens identified as *U*. *procera* (N = 30) in the *tuf*A phylogeny appeared as *U*. *linza* in the ITS phylogeny (Figs 3 and 4).

For the ITS marker, estimates of average intraspecific divergence ranged from 0% [*U*. *arasakii* (N = 11), *U*. *australis* (N = 77), *U*. *flexuosa* (N = 8), *U*. *pertusa* (N = 2), *U*. *torta* (N = 5), and *U*. *californica* (N = 14)] to 0.9% [*U*. *ohnoi* (N = 14)], whereas the largest interspecific divergence was 9.8% between *U*. *mutabilis* and *U*. *reticulata* for all species analyzed in the ITS phylogeny. Lack of interspecific divergence (0%) was found between *U*. *americana* and *U*. *laetevirens*, *U*. *mutabilis* and *U*. *pseudocurvata*, *U*. *laetevirens* and *U*. *scandinavica*, *U*. *clathratioides* and *U*. *torta*, *U*. *australis* and *U*. *pertusa*, and *U*. *americana* and *U*. *scandivnavica*.

Additionally, eight specimens showed discordant species identities between two different gene trees. Three specimens were identified as *Ulva* species in one marker and *Blidingia* spp. in the other marker (U109: *U*. *procera* vs. *Blidingia* sp.; U197: *Blidingia* sp. vs. *U*. *arasakii*; U213: *U*. *ohnoi* vs. *Blidingia* sp.). Five specimens were identified as different *Ulva* species between the phylogenies (U050, 391: *U*. *ohnoi* vs. *U*. *australis*; U075: *U*. *australis* vs. *U*. *spinulosa*; U225: *U*. *torta* vs. *U*. *linza*; U382: *U*. *australis* vs. *U*. *compressa*).

Considering a combined analysis of two phylogenies based on three different methods (NJ, ML, and BI) and interspecific sequence divergence estimates, nine *Ulva* species were finally identified from the 215 collected specimens (Table 1). The three most abundant species were *U*. *australis* (= *U*. *pertusa*) (34.4%, N = 74), *U*. *ohnoi* (*U*. *spinulosa* for ITS) (21.9%, N = 47), and *U*. *procera* (*U*. *linza* for ITS) (14.0%, N = 30) that were observed from 16, 16, and 11 out of the 23 sampling sites, respectively. The least abundant species included *U*. *compressa* (1.9%, N = 4), *U*. *laetevirens* (1.9%, N = 4), and *U*. *torta* (1.9%, N = 4), which were all detected from 2 of the 23 sites. Only 58 specimens (27%) were matched with species identification based on morphology by eye when collected in the field (S1 Table). Even 14 specimens identified as *Blidingia* spp. in the phylogeny were recognized as *Ulva* species at the time of collection.

### Model-based species delimitation analyses

The number of *Ulva* species identified by phylogenetic analyses lied within the range of the number of species groups or clusters estimated from the ABGD and GMYC analyses. In ABGD analyses, the number of species groups varied depending on *P* and X values. The number of groups identified ranged from one (when *P* = 0.1000) to 53 (when *P* = 0.0010) for *tuf*A, while from one (when *P* = 0.1000) to 52 (when *P* = 0.0010) for ITS from the whole data set (215 specimens) (S2 Table). The number of groups, showing a minimum difference (i.e., one) between the results of the initial partition (IP) and recursive partition (RP), was 17 (IP) and 18 (RP) for the *tuf*A gene dataset (when *P* = 0.0077, X = 1.5). Similarly, ITS dataset suggested 22 (IP) and 23 (RP) groups of the minimum difference between two partitions (when *P* = 0.0129, X = 1.5) (Table 2; S2 Table). The number of groups was reduced by excluding *Blidingia* spp. and unidentified species (Table 2). Given the minimum difference in the group number between IP and RP (i.e., one), number of groups only for *Ulva* specimens was suggested as six for *tuf*A (when *P* = 0.0077, X = 1.5) and eight for ITS (when *P* = 0.0129, X = 1.5) (Table 2).

GMYC model was not favored over the null model for *tuf*A (*P* = 0.15) and ITS (*P* = 0.42), which implies a wide range of confidence interval (95% CI) in the number of Maximum Likelihood (ML) clusters from one to 14 for *tuf*A and from one to seven for ITS (S2 Table). GMYC single-threshold analyses generated 13 clusters from *Ulva* species for *tuf*A, whereas only three clusters were identified in ITS dataset. Non-sampled species, which included only from the GenBank database, were identified as four clusters. All collected *Ulva* specimens analyzed in this study were finally grouped into nine *Ulva* species clusters, which was consistent with the findings of the species identification based on the phylogenetic analyses (Table 2; Fig 2; S3 Table).

## Discussion

We find a total of 9 *Ulva* species and *Blidingia* spp. distributed across 23 sites in Jeju Island, based on a combined analysis of cpDNA *tuf*A and nuDNA ITS-based phylogenies. *Tuf*A gene appears to be a more appropriate marker in differentiating nine different *Ulva* species relative to ITS region since the ITS phylogeny could not provide enough resolution or high support for several species groups, such as *U*. *ohnoi* with other closely related species and *U*. *australis* itself (Figs 3 and 4). Furthermore, ITS marker in GMYC analyses suggested three clusters of *Ulva* species (Table 2) and could not recover further distinct groups, which was clearly found as a monophyly (e.g., *U*. *torta*) in both phylogenetic trees (Figs 3 and 4).

Seven of nine *Ulva* species were identified as the same species based on two different phylogenies, whereas two species, *U*. *ohnoi* and *U*. *procera*, determined in the *tuf*A phylogeny were identified as *U*. *spinulosa* and *U*. *linza*, respectively in the ITS phylogeny (Figs 3 and 4). *Ulva ohnoi* complex as we found in the ITS phylogeny (Fig 4) has been repeatedly reported in previous studies [31, 32]. Recent evidence for ongoing speciation between *U*. *ohnoi* and *U*. *reticulata* (which belongs to the *U*. *ohnoi* complex) through postzygotic isolation has been documented [33]. Although we could not determine distinct species precisely for the 47 specimens in the ITS phylogeny (Table 1), *U*. *ohnoi* or *U*. *ohnoi* complex including *U*. *spinulosa* would be acceptable based on the *tuf*A phylogeny as well as the lowest level of average sequence divergence (0.5%) detected between *U*. *ohnoi* and *U*. *spinulosa*. Distinct species delimitation for *U*. *procera* and *U*. *linza* has also been long debated under *Ulva linza-procera-prolifera* (LPP) complex, which has been suggested by morphological and molecular analyses [31, 34, 35]. Thus, we regarded the 30 specimens showing a *U*. *procera*–*U*. *linza* discordance between two phylogenies as a single species group instead of two distinct species (Table 1).

Our analyses reveal that three species, *U*. *australis* (34% of 215 samples; 70% of 23 sites detected), *U*. *ohnoi* (22%; 70%), and *U*. *procera* (14%; 48%) are the predominant species along the Jeju Island coast. The occurrences of 5 species, *U*. *compressa*, *U*. *flexuosa*, *U*. *laetevirens*, *U*. *torta*, and *U*. *arasakii* in Jeju Island are first reported in this study, although they have previously been recorded from the mainland [3, 4, 10]. The two species, *U*. *procera* and *U*. *flexuosa*, have been designated as introduced species by the Ministry of Oceans and Fisheries of the Korean government [36]. Reference sequences retrieved from the GenBank database for *U*. *procera* whose type locality is Sweden [37] were obtained from the specimens collected from Canada: New Brunswick (HQ610393), Australia: Victoria (JN029336), and USA: California (KM255014). Also *U*. *flexuosa* having a type locality of the Mediterranean Sea [38] was used for the reference sequences from the specimens collected from Australia: New South Wales (JN029308) and Canada: British Columbia (HQ610296). A recent molecular study of the type specimen of *U*. *australis* suggests that its introduction from northeastern Asia (e.g., Japan) to Australia occurred via direct shipping by the middle of 19^th^ century [39]. Thus, dispersal of the two species, *U*. *procera* and *U*. *flexuosa* from those regions (e.g., Europe, North America, Australia) to Jeju Island is plausible via transportation of ballast waters, as seen for *U*. *ohnoi* and *U*. *fasciata* (*U*. *lactuca* [30]) between Japanese waters and the Mediterranean Sea [40] and *U*. *australis* between Japanese waters and Australia [39].

We find that there are no sites where all 9 *Ulva* species co-occurred and no more than four species were observed within each site (Table 1; Fig 2). However, more abundant species tended to distribute geographically more widely. These findings indicate that some species, such as *U*. *australis*, *U*. *ohnoi*, and *U*. *procera*, are predominant and widely distributed across the Jeju Island coast. *Ulva australis* is known to be the most common species involved not only macroalgal assemblages, but also green tides in Korea and also in Japan [41, 42]. This species is massively recruited during the late summer-autumn season and has sheet-like blades enabling high surface area [SA] to volume [V] ratio, which is favorable for absorbing inorganic nutrients in water columns [13]. The growth rate of *U*. *australis* is facilitated by high inorganic nitrogen concentrations (NH_4_^+^ and NO_3_^-^ + NO_2_^-^) in the surface water [43], and this species remains in a relatively high biomass during the winter period [42]. These characteristics can allow *U*. *australis* to last for more than five months after a bloom [13]. Thus, *U*. *australis* out-competes other *Ulva* species for space and nutrients. *Ulva ohnoi* is the most commonly-recorded species for green tides in various geographical regions [31, 32, 44–46]. This species shows a high affinity for nutrients, a high growth rate, and a broad range of tolerance to environmental conditions [45, 47, 48]. In particular, *U*. *ohnoi* shows high growth rates even in high water temperatures during summer as this species has a subtropical or tropical origin [31, 49]. This species has huge free-floating blades of approximately 4.0 m length by 4.0 m width (SRP, personal observation) suitable for fast uptake of nutrients. Seasonal succession of dominance between *U*. *australis* (or other *Ulva* species) and *U*. *ohnoi* might occur along the coasts of Jeju Island [46, 50], suggesting the lasting of serious local green algal massive growth over the summer period.

*Ulva linza* (*U*. *procera* for *tuf*A) is a representative green-tide forming macroalgae in Korea, China, and Japan [7, 12, 51]. In contrast to *U*. *australis* and *U*. *ohnoi*, *U*. *linza* has thin and tubular blades (low SA to V ratio), which helps to mitigate wave-induced stresses [13]. *Ulva linza* shows rapid growth rates under low salinity and high nutrient conditions [13, 48]. Due to its ecological and physiological characteristics, initial blooms of *U*. *linza* occur in brackish waters and subsequently move from estuaries into offshore areas [52]. Accordingly, *U*. *linza* can be an important source for more serious green-tide blooms.

The species diversity and composition that we observed in Jeju Island might be attributed to species’ ecological characteristics such as survival, habitat, growth rate, and adaptive capacity (e.g., responding to environmentally stressful conditions such as salinity and desiccation) [53, 54]. Less abundant species such as *U*. *compressa*, *U*. *laetevirens*, and *U*. *torta* might have lower growth rates or weaker competitive abilities (e.g., nutrient uptake rate) compared with others. Worldwide, *U*. *compressa* occurring in brackish environments or in the upper intertidal zone may have adapted to low salinity and high desiccation [7, 12, 55–57]. As shown in previous studies, this species is found in only two sites with an extensive intertidal zone in this study. However, *U*. *compressa* would show low biomass and coverage in the field as this species persists for less than two months and is exposed to high herbivore pressure due to its morphology [13].

Less abundant species such as *U*. *compressa*, *U*. *laetevirens*, and *U*. *torta* were observed only at sites in the proximity of the harbor in this study. This implies that they might be recently introduced to Jeju Island from elsewhere, and there has been insufficient time for them to reach population stability there. This hypothesis is plausible, given that this is the first report of those three species occurring in Jeju Island. Although a total of 17 species of *Ulva* have been known in the marine algal flora of Korea [2, 4], the existence of only 7 species: *U*. *pertusa*, *U*. *linza* (*U*. *procera*), *U*. *fasciata* (currently identified as *U*. *lactuca* [30]), *U*. *californica*, *U*. *conglobata*, *U*. *lactuca* (currently identified as *U*. *fenestrata* [30]), and *U*. *japonica* (currently named as *Umbraulva japonica* [58]) have been reported in Jeju Island to date [3, 10]. Recently, *U*. *torta* whose type locality is Germany [38, 59] was first reported on the eastern coast of Korea [1]. We further demonstrate its habitation on the coast of Jeju Island, albeit relatively rare.

Alternatively, different species composition and varying extents of species diversity among the 23 sites might be partly explained by the patterns of green algal blooming in the Yellow Sea in the northwestern parts of Jeju Island. *Ulva* species are major macroalgae leading to green-tide blooming, especially around the northwestern Pacific regions, including China, Japan, and Korea [10, 60–62]. In particular, Jeju Island is one of the places suffering from severe green tides every year in Korea. Free-floating species, such as *U*. *compressa*, *U*. *flexuosa*, and LPP (*linza-procera-prolifera*) complex have been suggested to be main species responsible for the Yellow Sea green tides [63–65]. We found that the third most common species in Jeju Island was *U*. *procera* (*U*. *linza*). This species has been identified as a key species causing green tides in the open water near the southwestern coasts of Korea, which is the southeastern part of the Yellow Sea green-tide patch [10]. Thus, the Yellow Sea green tides might possibly lead to the observed relatively high proportions of *U*. *procera* on the Jeju Island coast. However, this hypothesis needs to await future research.

The species diversity and composition in the green-tide *Ulva* communities on Jeju Island might vary in space and time. It has been reported that the most common species changes from *U*. *pertusa* in spring and summer to *U*. *ohnoi* in winter in Mikawa Bay, Japan [50]. The study specimens were sampled during a spring-summer season, and therefore further investigations with additional samples collected at a different season (e.g., fall-winter) will allow us to understand seasonal changes in dominant *Ulva* species and thus help to predict the intensity and range of local green tides on Jeju Island in the future.

The largest degrees of interspecific genetic distances for *tuf*A (11%) and ITS (9.8%) genes found in this study are within the previously reported ranges (2.1%–13.3%) of *Ulva* species, as suggested in [10]. Moreover, we found less than 1% average interspecific divergence in several species pairs, such as between *U*. *ohnoi* and *U*. *fasciata* (currently identified as *U*. *lactuca* [30]) (0.7%) in *tuf*A, as suggested in a previous study [66]. Interspecific distance in *tuf*A between *U*. *laetevirens* and *U*. *rigida* showed 0% average sequence divergence. For some species, we have considered them as different species rather than synonyms based on our phylogenetic tree-based criteria (see above). For example, *U*. *flexuosa* and *U*. *californica* are considered as different species, since they form separate monophyletic groups despite the low divergence of 1% and 0.7% for *tuf*A and ITS, respectively. In GMYC analyses, these two species were clustered separately for *tuf*A while the same cluster for ITS (Table 2). However, the species delimitation (including taxonomical synonym) for *Ulva* should be treated with caution, particularly when a few molecular markers show identical sequences or very low levels of sequence divergence between the study objects. Recent whole-mitogenome sequencing of *U*. *pertusa*, known as a taxonomic synonym of *U*. *australis* [67], showed distinct interspecific variation, such as 4.7 kb size difference and variable tandem duplication mutations. Thus, vigorous analysis at a large genomic scale is required to accurately define “true” species or taxonomical synonyms in the genus *Ulva*.

Our phylogenetic tree-based species delimitation for the genus *Ulva* using two different marker-sets reveals an identical number of species, although tree topologies of some species (e.g., *U*. *ohnoi−U*. *spinulosa*, *U*. *procera−U*. *linza*) are incongruent between the two phylogenies. Furthermore, our algorithmic species delimitation approaches, particularly GMYC supported the number of species groups for *Ulva*. These results highlight the reliability and accuracy of our tree-based criteria for species delimitation of *Ulva*, which has been shown to demonstrate taxonomic complexities, such as several cases of synonyms. Our own morphology-based species identification by eye in the field showed surprisingly high levels of misidentification, emphasizing the importance of molecular-based species determination. It is well known that valid taxonomical characters are difficult to apply to species identification for *Ulva*, due to the extremely high levels of phenotypic plasticity in response to various environmental factors [5, 6]. Nevertheless, detailed morphological assessments along with genetic analysis will provide intriguing insights into the interspecific differences in the levels of phenotypic diversity among different *Ulva* species. Our study shows that the molecular-based phylogenetic and model-based approaches using two different molecular markers (i.e., chloroplast and nuclear DNA) can be applied to efficient and reliable species identification for monitoring changes in species composition and relative abundances of the green-tide *Ulva* masses in large coastal areas. Also, the approaches and the results reported in this study can help to assess fluctuations in species composition and seasonal population dynamics at the sites which are currently experiencing rapid environment changes, such as Jeju Island, Korea.

## Supporting information

S1 Table Detailed information for collection sites, specimen ID, and species delimitation based on six phylogenetic criteria (two genes × three different phylogenetic inferences). (CSV)
(XLSX)

S2 Table Results of partition by ABGD analyses for *tuf*A and ITS markers. *P*, Prior intraspecific divergence (*P*); IP, Initial partition; RP, Recursive partition; *N*_*g*_, Number of groups of the full data set (*N*_*g*_).
(XLSX)

S3 Table Results of the GMYC analyses based on the *tuf*A and ITS marker. (XLSX)
